# Antidepressant Sertraline Synergistically Enhances Paclitaxel Efficacy by Inducing Autophagy in Colorectal Cancer Cells

**DOI:** 10.3390/molecules29163733

**Published:** 2024-08-07

**Authors:** Leping He, Yuxi Tian, Qingqing Liu, Jiaolin Bao, Ren-Bo Ding

**Affiliations:** 1 Key Laboratory of Tropical Biological Resources of Ministry of Education, School of Pharmaceutical Sciences, Collaborative Innovation Center of One Health, Hainan University, Haikou 570228, China; hlprin98@126.com (L.H.); a18280572994@163.com (Y.T.); qingqing200104@163.com (Q.L.); baojiaolin@hainanu.edu.cn (J.B.); 2 State Key Laboratory of Quality Research in Chinese Medicine, Institute of Chinese Medical Sciences, University of Macau, Macao 999078, China

**Keywords:** colorectal cancer, sertraline, paclitaxel, autophagy, drug combination

## Abstract

Colorectal cancer (CRC) is the second leading cause of cancer-related death worldwide. It is important to discover new therapeutic regimens for treating CRC. Depression is known to be an important complication of cancer diseases. Repurposing antidepressants into anticancer drugs and exploring the combinational efficacy of antidepressants and chemotherapy are potentially good options for developing CRC treatment regimens. In this study, sertraline, an antidepressant drug, and paclitaxel, an anticancer drug, were chosen to study their antitumor effects in the treatment of colorectal cancer, alone or in combination, and to explore their underlying mechanisms. The data showed that sertraline exerted a dose-dependent cytotoxic effect on MC38 and CT26 colorectal cancer cell lines with IC_50_ values of 10.53 μM and 7.47 μM, respectively. Furthermore, sertraline synergistically sensitized chemotherapeutic agent paclitaxel efficacy in CRC cells with combination index (CI) values at various concentrations consistently lower than 1. Sertraline remarkably augmented paclitaxel-induced autophagy by increasing autophagosome formation indicated by elevated LC3-II/I ratio and promoting autophagic flux by degrading autophagy cargo receptor SQSTM1/p62, which may explain the synergistically cytotoxic effect of sertraline and paclitaxel combination therapy on CRC cells. This study provides important evidence to support repurposing sertraline as an anticancer agent and suggests a novel combinational regimen for effectively treating CRC as well as in the simultaneous treatment of CRC and depression.

## 1. Introduction

Cancer is a major global public health problem, and colorectal cancer (CRC) is the third most commonly diagnosed cancer and the second leading cause of cancer-related death worldwide [1,2]. Major depressive disorder is known to be an important complication of cancer diseases, with a three-fold higher incidence in cancer patients than in the healthy population [3]. Cancer-associated depression increases the mortality rate of cancer patients greatly, varying from 26% to 39%, depending on the degree of depressive symptoms [4]. Therefore, antidepressants are commonly used as a complementary regimen during cancer therapy.

A series of studies have demonstrated that antidepressants exhibited anticancer properties against various types of cancer [5]. Their anticancer pharmacological mechanisms include inducing apoptosis, suppressing proliferation, inhibiting tumor angiogenesis, regulating cellular metabolism, modulating the antitumor immune system, and so on [6,7,8]. In recent years, autophagy regulation has been considered a new mechanism responsible for the antidepressant-associated anticancer effect [5].

Sertraline is the most prescribed antidepressant drug in North American markets, which is a type of selective serotonin reuptake inhibitor (SSRI). Sertraline has been reported to exert anticancer effects among lung cancer [9], hepatocellular carcinoma [10], and breast cancer [11]. Paclitaxel is one of the most commonly used anticancer drugs for CRC treatment. However, few studies have investigated the effects of the combination of antidepressants and anticancer drugs on cancer treatment and whether it is synergistic or antagonistic. It is extremely urgent to understand this aspect since simultaneous treatment of anticancer drugs and antidepressant drugs has already been widely used for cancer patients accompanied by depression in clinical. In this study, sertraline, one of the most prescribed antidepressant drugs, and paclitaxel, one of the most used anticancer drugs for CRC treatment, were chosen to study the effects of their combination in the treatment of colorectal cancer and to explore the underlying mechanisms.

## 2. Results

### 2.1. Effect of Sertraline and Paclitaxel on the Viability of CRC Cell Lines

**Figure 1:**
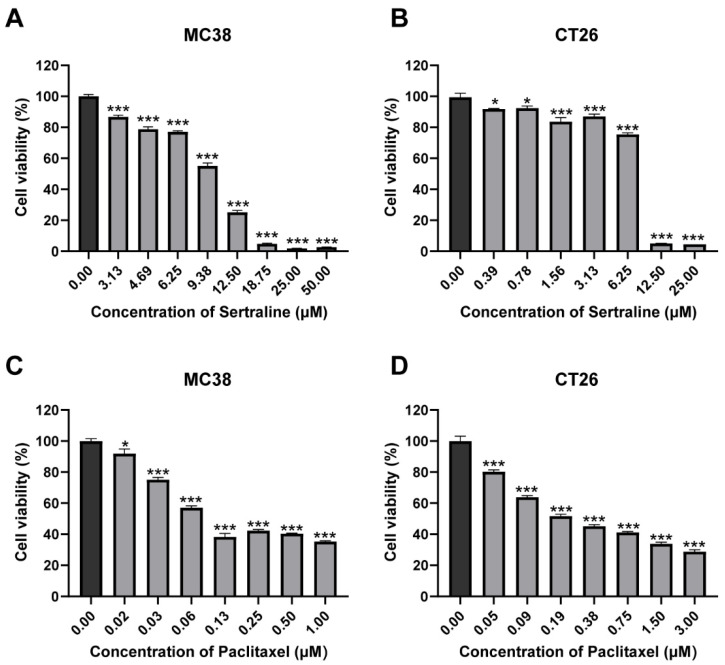
Sertraline and paclitaxel inhibited CRC cell growth in vitro. MC38 (**A**) and CT26 (**B**) cells were treated with gradient concentrations of sertraline for 72 h, and cell viabilities were measured by MTT assay. The cytotoxic effect of paclitaxel in MC38 (**C**) and CT26 (**D**) for 72 h. The values are expressed as mean ± SEM of 6 determinations. The statistical significance was calculated using one-way ANOVA by comparing the drug treatment group with the vehicle control group, * *p* < 0.05; *** *p* < 0.001.

The cytotoxic effects of sertraline and paclitaxel were investigated by incubating CRC cell lines with varying concentrations of each drug for 72 h. The results of the MTT assay revealed that sertraline exerted a dose-dependent cytotoxic effect on MC38 and CT26 cell lines, and their IC_50_ values were 10.53 μM and 7.47 μM, respectively, which indicated a significant reduction in cell viability by approximately 50% (*p* < 0.05) (Figure 1A,B). Similarly, paclitaxel demonstrated remarkable cytotoxicity in a dose-dependent manner against these cell lines (*p* < 0.05) (Figure 1C,D). The cell viability (%) graphs obtained from these results are presented in Figure 1. Statistical analysis using ANOVA confirmed significant differences in mean values among the groups.

### 2.2. Sertraline Synergistically Sensitized Paclitaxel Efficacy against CRC Cells

**Figure 2:**
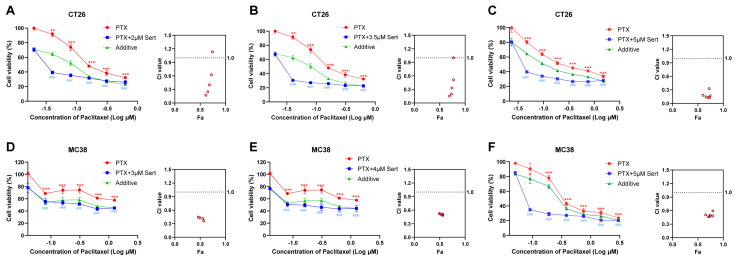
Sertraline synergistically enhanced paclitaxel efficacy in CRC cells. The combinational cytotoxic effect of sertraline and paclitaxel in CT26 cells (**A**–**C**) and MC38 (**D**–**F**) cells for 72 h. The blue line shows the actual drug-effect curves of drug combinations. The green line indicates the additive curves of drug combinations by calculation. Data were presented as the means ± SEM. The statistical significance was calculated using one-way ANOVA by comparing the drug treatment group with the vehicle control group; ** (paclitaxel treatment vs. control) *p* < 0.01, *** (paclitaxel treatment vs. control) or ^###^ (combinational treatment vs. control) *p* < 0.001. The combination index (CI) and Faction affected (Fa) values were calculated using CalcuSyn software (Version 2; Biosoft).

Depression is a prevalent and serious complication associated with cancer, often necessitating the use of antidepressants in clinical practice. Sertraline, a commonly prescribed antidepressant, has thus been widely administered alongside cancer therapies. In the present study, we explored the potential of sertraline to enhance the anticancer efficacy of paclitaxel, a standard chemotherapeutic agent, against CRC cells. The results indicated that the combination of sertraline and paclitaxel significantly reduced cell viability of CRC cells more effectively than paclitaxel treatment alone (Figure 2). Notably, the actual drug-effect curves (blue) of the sertraline and paclitaxel combination were remarkably lower than the predicted additive curves (green), which indicated a synergistic drug effect between these two drugs.

To quantitatively evaluate the synergy between sertraline and paclitaxel, we employed CalcuSyn software (Version 2; Biosoft, Acropolis Computers, Inc., Saint Louis, MO, USA) to calculate the combination index (CI). As shown in Table 1, the results revealed that the combination of sertraline and paclitaxel exerts a synergistically cytotoxic effect on CT26 and MC38 cells, with CI values at various concentrations consistently lower than 1. In particular, the sertraline and paclitaxel combination demonstrated a strong synergism in CT26 cells at various concentrations with CI values ranging from 0.1 to 0.3 [12]. These data supported the hypothesis that sertraline could synergistically sensitize the anticancer activity of paclitaxel against CRC cells.

### 2.3. Sertraline Augmented Paclitaxel-Induced Autophagy in CRC Cells

**Figure 3:**
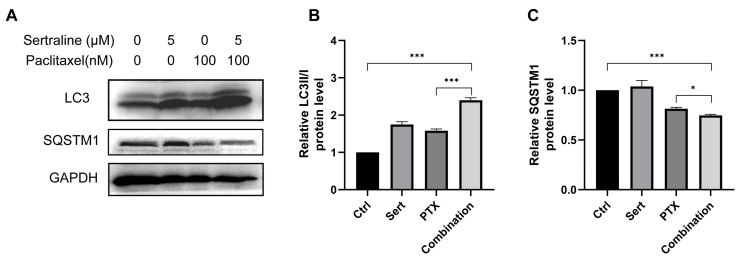
Sertraline augmented paclitaxel-induced autophagy. (**A**) Representative Western blot images showing the expression levels of LC3 and SQSTM1 in CT26 cells treated with 5 μM sertraline and 100 nM paclitaxel for 72 h. The densitometric analysis of LC3II/I ratio (**B**) and SQSTM1 (**C**). The statistical significance was calculated using an unpaired *t*-test by comparing indicated groups, * *p* < 0.05; *** *p* < 0.001.

Recent studies showed that paclitaxel could induce autophagic cell death in cancer cells [13,14]. Thus, we wondered if the enhanced anticancer effect of paclitaxel by sertraline treatment was associated with increased autophagy induction. Building on this, our study explores the combined effect of sertraline and paclitaxel on autophagy induction in CRC cells. A widely used approach to monitor autophagy is to detect the conversion of microtubule-associated protein light chain 3 (LC3) (LC3-I to LC3-II) and the degradation of autophagy cargo receptor, the protein sequestosome 1 (SQSTM1/p62), by immunoblot analysis [5]. We assessed the formation of LC3-II, a key marker of autophagy, in CT26 cells using Western blotting assays. Both 5 μM sertraline and 100 nM paclitaxel alone were able to prompt autophagy in CT26 cells, as evidenced by increased LC3-II levels. Notably, the combination of the two drugs resulted in a significantly enhanced LC3-II accumulation compared to either drug alone (Figure 3). Furthermore, SQSTM1/p62, an indicator of autophagic flux and degradation process, was also examined by Western blotting assays. The results showed that combinational treatment remarkably decreased the SQSTM1/p62 levels (Figure 3), confirming the enhanced autophagic activity. These results suggest that sertraline synergistically augmented paclitaxel-induced autophagy in CRC cells, potentially contributing to the exacerbated CRC cell death.

## 3. Discussion

Colorectal cancer (CRC) is the second most common cancer-related death [15]. About one-third of CRC patients would experience treatment failure when receiving conventional chemotherapy cycles [16,17]. Tumor heterogeneity is one of the key reasons responsible for treatment failure and tumor recurrence whenever fractions of tumor cells survive chemotherapy [18,19]. Combinational therapy that eliminates all tumor cell populations at once is considered an effective strategy to overcome potential cancer treatment failure. Therefore, it is necessary to develop new combinational therapy regimens for CRC patients, especially those that kill tumor cells, in addition to conventional mechanisms, such as induction of autophagic cell death. However, the process of developing a novel anticancer drug from the beginning is extremely expensive and time-consuming, which normally costs around USD 650 million per drug and takes an average of 17 years [20,21]. Thus, repurposing existing clinical drugs into new anticancer agents is a feasible and cost-effective option with a shortened development process.

Depression is known to be a frequently occurring complication of cancer, and evidence has shown that advanced CRC patients are more likely to suffer from major depressive disorder [22]. A series of studies have revealed that comorbid depression impairs prognosis and increases mortality in cancer patients [23,24,25]. If an antidepressant could have dual efficacy with an anticancer effect in addition to treating depression, it would be an ideal option for the development of cancer combinational therapy regimens.

Sertraline is an antidepressant drug of selective serotonin reuptake inhibitor, which blocks the pre-synaptic serotonin reuptake transporter and accumulation of serotonin at the synaptic cleft [26]. Sertraline is the most prescribed antidepressant drug in the United States and has demonstrated good medication safety [27]. Besides its benefits in the treatment of psychiatric disorders, sertraline was reported to have good anticancer activity in various types of cancers [5,28]. The induction of apoptotic cell death and suppression of tumor cell proliferation are considered the predominant mechanisms responsible for sertraline-associated anticancer effects [11,29,30,31,32,33,34]. The current study showed that sertraline exerted effective anticancer activity in CRC cells (Figure 1), which provides further evidence to support repurposing sertraline as an anticancer agent. In addition to possessing good anticancer effects when used alone, sertraline was also reported to have the potential to sensitize chemotherapeutic agents [5,28]. The current study demonstrated that sertraline synergistically enhanced paclitaxel efficacy against CRC (Figure 2), which suggests a novel combinational regimen for effectively treating CRC as well as in the simultaneous treatment of CRC and depression.

Autophagy is an evolutionarily conserved cellular process that functions to degrade and recycle dysfunctional proteins and organelles in autolysosomes [35,36,37]. Autophagy is activated by AMPK-mTOR signaling, which is a crucial energy sensor in the maintenance of cellular energy homeostasis. In nutrient-limitation conditions, autophagy normally functions as a protective mechanism to promote cell survival by recycling essential cellular components [38]. However, when autophagy is excessively induced, the programmed autophagic cell death would occur [39,40,41]. Accumulating evidence has uncovered the importance of autophagy in regulating cancer, and targeting autophagy-associated programmed cell death to develop pharmacological autophagy inhibitors is considered a novel anticancer drug development strategy [42,43,44]. The current study showed that sertraline remarkably augmented paclitaxel-induced autophagy by increasing autophagosome formation indicated by elevated LC3-II/I ratio and promoting autophagic flux by degrading autophagy cargo receptor SQSTM1/p62 (Figure 3), which may explain the synergistically cytotoxic effect of sertraline and paclitaxel combination therapy on CRC cells. Therapeutic strategies that target autophagy induction may represent an effective approach for enhancing anticancer efficacy in the treatment of CRC.

There are still some unsolved works to complete in the future before the wide application of sertraline and paclitaxel combination for clinically treating CRC patients. Firstly, more in vivo and clinical trial investigations are required to further demonstrate the efficacy and safety with solid evidence. Secondly, more intensive mechanism studies should be further conducted to elucidate the underlying pharmacological and pharmacokinetic actions of sertraline and paclitaxel combination, especially the drug interaction effects on ADMET (absorption, distribution, metabolism, excretion, and toxicity).

## 4. Materials and Methods

### 4.1. Cell Culture

The colorectal cancer (CRC) cell lines CT26 and MC38 were purchased from Procell (Wuhan, China), cultured in a humidified incubator at 37 °C in an atmosphere of 5% CO_2_, and maintained in RPMI-1640 (PM150110, Procell) supplemented with 10% fetal bovine serum (FBS) and 1% Penicillin–Streptomycin solution (PB180120, Procell). The cell lines were authenticated and regularly tested for mycoplasma contamination.

### 4.2. Antibodies and Reagents

Antibodies against LC3 (1:1000, 14600-1-AP), SQSTM1/p62 (1:1000, 18420-1-AP), and GAPDH (1:10,000, 10494-1-AP) were purchased from Proteintech (Wuhan, China). Sertraline hydrochloride (S129789) and Paclitaxel (P106869) were purchased from Aladdin (Shanghai, China). The MTT (ST1537) and lysis buffer (P0013J) were purchased from Beyotime (Shanghai, China).

### 4.3. Cell Viability Assay

The MTT assay was conducted to evaluate the cell viability of CRC cell lines in response to sertraline and paclitaxel. Cells were plated at a density of 4000 cells/well in 96-well plates and allowed to adhere overnight. Subsequently, various concentrations of sertraline and paclitaxel were administered to the cells, and they were incubated for 72 h. Cell viability was then assessed by adding 5 mg/mL MTT solution to each well and incubating for 4 h to allow for formazan crystal formation. The medium was carefully removed, and the formazan crystals were solubilized in 100 μL DMSO. The absorbance was measured at 570 nm to determine the relative number of viable cells.

### 4.4. Western Blot Analysis

Cell lysates were prepared following incubation in lysis buffer supplemented with PMSF (P0100, Solarbio, Beijing, China) and phosphatase inhibitors (4906837001, Roche, Basel, Switzerland). The cells were lysed for 30 min on ice before being centrifuged at 12,500 rpm for 20 min at 4 °C. Protein concentrations in the supernatants were determined using a Bicinchoninic acid (BCA) assay (AR1189, Boster Biotechnology, Wuhan, China). Equal amounts of protein (20 μg) from each lysate were resolved on 15% SDS-PAGE for separation, then electrotransferred onto PVDF membranes. Western blot analysis was performed by incubating PVDF membranes with appropriate primary and secondary antibodies overnight at 4 °C and 2 h at room temperature, respectively. The protein bands were visualized using an ECL chemiluminescence detection kit (AR1172, Boster Biotechnology).

### 4.5. Determination of Synergistic Effect and Additive Effect

A synergistic effect occurs when two or more drugs (or chemicals) combine to produce a greater effect than the sum of the effects if each drug were given separately. An additive effect occurs when two or more drugs (or chemicals) combine and produce a total effect equal to the sum of the effects of each individual drug. The dose–response effects of sertraline and paclitaxel were determined for selecting the base drug and its dosage for use in drug combination treatment. A dose of the base drug (sertraline) not higher than the IC_50_ value was recommended. To calculate the theoretical additive effect of sertraline and paclitaxel combination, the following formula was used [19,45]:

E_total_ = E_1_ + E_2_ − E_1_ × E_2_

E_1_ is the inhibitory effect of sertraline at a dose of 5 μM, and E_2_ is the inhibitory effect of paclitaxel at various doses.

The fitting curve of dose–response effect of paclitaxel treatment alone, actual combinational treatment, and theoretical additive effect were drawn, which could classify a synergistic drug combination if the actual dose–response curve of combinational treatment is lower than the additive effect curve.

Combination index (CI) values can be used to quantitatively evaluate the synergy between sertraline and paclitaxel. CalcuSyn software or CompuSyn online tool can be employed to calculate CI values by input drug doses used and the corresponding inhibitory effect of the drug according to the manual instruction. This classifies a synergistic drug combination if the CI value is lower than 1. More specifically, CI = 0.85–0.90 indicates slight synergism, CI = 0.70–0.85 indicates moderate synergism, CI = 0.3–0.7 indicates synergism, CI = 0.1–0.3 indicates strong synergism, CI < 0.1 indicates very strong synergism, CI = 1.10–1.20 indicates slight antagonism, CI = 1.20–1.45 indicates moderate antagonism, CI = 1.45–3.3 indicates antagonism, CI = 3.3–10 indicates strong antagonism, CI > 10 indicates very strong antagonism [12].

### 4.6. Statistical Analysis

All data sets, derived from three independent experiments, were subjected to statistical analysis using one-way ANOVA with multiple comparisons or unpaired *t*-tests, where significance was set at a 5% threshold. The results are expressed as mean ± SEM, and all analyses were performed using GraphPad Prism 8 (ver.8.3.0 for Windows; GraphPad Software, Inc., San Diego, CA, USA). A *p*-value < 0.05 was considered statistically significant.

## 5. Conclusions

The present study demonstrated that sertraline exerted effective anticancer activity in CRC cells and sertraline synergistically enhanced the efficacy of the chemotherapeutic agent paclitaxel against CRC. Sertraline remarkably augmented paclitaxel-induced autophagy by increasing autophagosome formation indicated by elevated LC3-II/I ratio and promoting autophagic flux by degrading autophagy cargo receptor SQSTM1/p62, which may explain the synergistically cytotoxic effect of sertraline and paclitaxel combination therapy on CRC cells. This study provides further evidence to support repurposing sertraline as an anticancer agent and suggests a novel combinational regimen for effectively treating CRC as well as in the simultaneous treatment of CRC and depression.

## Figures and Tables

**Table 1:** Combination indexes of paclitaxel and sertraline in CT26 and MC38.

Cell Lines	Sertraline (μM)	Paclitaxel (μM)	CI Value	Description
CT26	5	0.05	0.18033	Strong synergism
		0.09	0.14366	Strong synergism
		0.19	0.1306	Strong synergism
		0.38	0.12969	Strong synergism
		0.75	0.1653	Strong synergism
		1.50	0.32759	Synergism
	3.5	0.04	0.15255	Strong synergism
		0.08	0.19962	Strong synergism
		0.16	0.33053	Synergism
		0.31	0.51391	Synergism
		0.63	1.00122	Nearly additive
	2	0.04	0.17338	Strong synergism
		0.08	0.24892	Strong synergism
		0.16	0.40078	Synergism
		0.31	0.62531	Synergism
		0.63	1.13285	Nearly additive
MC38	5	0.09	0.50569	Synergism
		0.19	0.46353	Synergism
		0.38	0.46892	Synergism
		0.75	0.49670	Synergism
		1.5	0.48363	Synergism
		3.0	0.59361	Synergism
	4	0.08	0.52681	Synergism
		0.16	0.51492	Synergism
		0.31	0.49307	Synergism
		0.63	0.48567	Synergism
		1.25	0.51280	Synergism
	3	0.08	3.28947	Antagonism
		0.16	0.44676	Synergism
		0.31	0.43279	Synergism
		0.63	0.36088	Synergism
		1.25	0.41475	Synergism

## Data Availability

Data will be made available on request.
